# Admixture Analysis Using Genotyping-by-Sequencing Reveals Genetic Relatedness and Parental Lineage Distribution in Highbush Blueberry Genotypes and Cross Derivatives

**DOI:** 10.3390/ijms22010163

**Published:** 2020-12-26

**Authors:** Krishnanand P. Kulkarni, Nicholi Vorsa, Purushothaman Natarajan, Sathya Elavarthi, Massimo Iorizzo, Umesh K. Reddy, Kalpalatha Melmaiee

**Affiliations:** 1 Department of Agriculture and Natural Resources, Delaware State University, Dover, DE 19901, USA; kkulkarni@desu.edu (K.P.K.); selavarthi@desu.edu (S.E.); 2 Department of Plant Biology, Rutgers University, New Brunswick, NJ 08901, USA; vorsa@njaes.rutgers.edu; 3 Philip E. Marucci Center for Blueberry and Cranberry Research and Extension, Chatsworth, NJ 08019, USA; 4 Department of Biology, West Virginia State University, Institute, WV 25112, USA; pnatarajan@wvstateu.edu (P.N.); ureddy@wvstateu.edu (U.K.R.); 5 Department of Horticultural Science and Plants for Human Health Institute, NC State University, Kannapolis, NC 28081, USA; miorizz@ncsu.edu

**Keywords:** *Vaccinium*, admixture analysis, genotyping-by-sequencing, genetic relatedness

## Abstract

Blueberries (*Vaccinium* section *Cyanococcus*) are perennial shrubs widely cultivated for their edible fruits. In this study, we performed admixture and genetic relatedness analysis of northern highbush (NHB, primarily *V. corymbosum*) and southern highbush (SHB, *V. corymbosum* introgressed with *V. darrowii, V. virgatum, or V. tenellum*) blueberry genotypes, and progenies of the BNJ16-5 cross (*V. corymbosum* × *V. darrowii*). Using genotyping-by-sequencing (GBS), we generated more than 334 million reads (75 bp). The GBS reads were aligned to the *V. corymbosum* cv. Draper v1.0 reference genome sequence, and ~2.8 million reads were successfully mapped. From the alignments, we identified 2,244,039 single-nucleotide polymorphisms, which were used for principal component, haplotype, and admixture analysis. Principal component analysis revealed three main groups: (1) NHB cultivars, (2) SHB cultivars, and (3) BNJ16-5 progenies. The overall fixation index (*F_ST_*) and nucleotide diversity for NHB and SHB cultivars indicated wide genetic differentiation, and haplotype analysis revealed that SHB cultivars are more genetically diverse than NHB cultivars. The admixture analysis identified a mixture of various lineages of parental genomic introgression. This study demonstrated the effectiveness of GBS-derived single-nucleotide polymorphism markers in genetic and admixture analyses to reveal genetic relatedness and to examine parental lineages in blueberry, which may be useful for future breeding plans.

## 1. Introduction

Blueberries (*Vaccinium corymbosum* section *Cyanococcus*) are perennial shrubs native to eastern North America but are widely cultivated for their edible fruit in several countries, including Canada, Europe, Australia, New Zealand, Chile, and Argentina [1]. The *Vaccinium* genus includes important cultivated species such as blueberry and cranberry. The United States is the world’s largest producer of blueberries [2].

Blueberry is a very high-value crop [3] that can thrive on acidic soils. Consumer demand for blueberries is at an all-time high; hence, its production around the world has quickly increased (http://www.fao.org/faostat, accessed November, 2020) primarily because of its health benefits [4]. Blueberries contain a large amount of antioxidant phenolic compounds, anthocyanins, flavonols, and phenolic acids. Several epidemiological studies associated regular small-to-moderate intake of blueberries with reduced risk of cardiovascular disease, cancer, obesity, and type 2 diabetes [5].

Blueberries were recently domesticated in the twentieth century [6], and breeding and genetic improvement started in 1909 [7] with the selection of clones from wild populations and cross-pollination, leading to breeding and selection cycles. Initial blueberry improvement efforts mainly focused on developing cultivars adaptable to the broader climatic conditions, and improving winter hardiness, fruit quality, and mechanical harvesting [1].

The *Vaccinium* genus includes approximately 450 species [8], and the blueberry germplasm include diploid (2n = 2× = 24), tetraploid (2n = 4× = 48) and hexaploid (2n = 2× = 72) species [9,10,11,12,13]. All species in *Vaccinium* sect. *Cyanococcus* are highly or mostly self-sterile, and diploids are essentially obligatory outcrossing [14,15,16]. Breeding largely at the tetraploid level has led to cultivars with earlier ripening berries, increased berry size, and higher fruit set [17]. Interspecific hybridization has played a crucial role in the development of cultivars with improved trait performance but has also led to complex relationships among blueberry species.

*V. angustifolium* is thought to be one of the first blueberry species used for fruit production in North America [18]. The highbush blueberry (*V. corymbosum*) is the major cultivated blueberry type in North America and the world [19]. Commercially grown cultivars of highbush contribute about two-thirds of the total production, while the remaining one-third comes from the lowbush (*V. angustifolium*) species. *V. corymbosum* cultivars with high chilling requirements (>800 chilling hours measured as accumulated hours of temperature <7 °C) for the initiation of flowering are called northern highbush (NHB) cultivars [20], and those with lower chilling requirements are called southern highbush (SHB) cultivars. The commercial SHB cultivars were developed from NHB cultivars by the introgression of genes from *V. darrowii*, *V. virgatum*, and *V. tenellum*, with *V. darrowii* being the largest contributor of genetic material [19]. There is evidence that *V. darrowii* is the most ancestral taxon of the *Cyanococcus* section [21] and *V. darrowii* may have played a greater role in the evolution of this section as a sole survivor of the extant taxa [14]. In this process, today’s cultivars represent a mixture of alleles from four different species. Such admixed populations complicate mapping endeavors of various loci governing complex traits. Hence, analyzing the admixture of genetic makeup of individuals is extremely important for association mapping and population genetic analyses [22]. Extant highbush cultivars result from the presence of the wide-ranging contribution of *V. corymbosum* genome combined with lineages of *V. darrowii*. Admixture analysis with a high-density single-nucleotide polymorphism set (SNPset) that is distributed across all chromosomes would help reveal lineage sorting among cultivar germplasm.

The use of high-density SNPs for genetic analysis research in blueberry has been limited until recent advancements in next-generation sequencing (NGS) technologies. Several studies have used older generation types of molecular markers in highbush blueberry for population structure analysis: random amplification of polymorphic DNA (RAPD), simple sequence repeats [6,19,23,24], expressed sequence tag (EST)-PCR markers [19,25,26], and retrotransposon-based sequence-specific amplification polymorphism markers [27]. However, such marker systems have several limitations and are not amenable for high-throughput screening of larger populations.

Genotyping-by-sequencing (GBS) is a reduced representation method that utilizes NGS and can be used to resolve population structure for use in genome-wide association studies (GWAS). Furthermore, increased marker density across the chromosomes facilitates linkage disequilibrium (LD) analysis and haplotype calling [28]. SNPs can be valuable in marker-assisted selection (MAS) to facilitate the introgression of traits into domesticated genetic backgrounds; such traits include aphid resistance from a diploid species *V. darrowii* [29] and unique fruit chemistry traits including fruit volatiles, organic acids, and flavonoids [30,31].

The objective of the present study was to (1) identify a large number of SNPs anchored to the genome sequence, and (2) utilize chromosome-specific SNP markers for admixture analysis and haplotype identification of the 99 blueberry accessions. The set included NHB and SHB cultivars, and F_1_ parents and F_2_ progeny derived from an interspecific diploid cross of the NHB genotype *V. corymbosum* adapted to a temperate climate with the evergreen blueberry genotype *V. darrowii* adapted to a subtropical climate. We also sought to characterize the LD patterns and perform haplotype block analysis. The findings in this study will be useful in future GWAS, MAS, and genetic characterization of blueberry species. 

## 2. Results

GBS with the 99 blueberry accessions generated more than 334 million reads (334,600,452) of 75 bp in length (Table S1). The average number of reads with tags per sample was 3.3 million, with a median of 3.4 million reads. Good barcoded tags with at least three read counts were used for SNP calling. The GBS reads were aligned to the *V. corymbosum* cv. Draper v1.0 reference genome sequence [32]. Details of the SNPs mapped to the longest 12 scaffold sequences of the Draper v1.0 genome are given in Table S2. An average of 2.8 million reads with a tag per sample were successfully mapped to the reference genome, which corresponds to an overall mapping rate of 83% to the genome. From the alignments, we identified 2,244,039 SNPs with the 99 selected accessions. The SNPs were filtered by using (1) read depth, DP < 3, (2) minor allele frequency (MAF) < 0.05, and (3) call rate < 0.9. After stringent SNP filtering, we obtained 92,048 SNPs distributed across the *V. corymbosum* reference genome, with an average of five SNPs per 1-kb genome length. The number of filtered SNPs mapped to the 12 scaffolds ranged from 6191 SNPs for VACCDSCAFF12 to 8994 for VACCDSCAFF2 (Table 1).

### 2.1. Principal Component Analysis (PCA)

**Figure 1:**
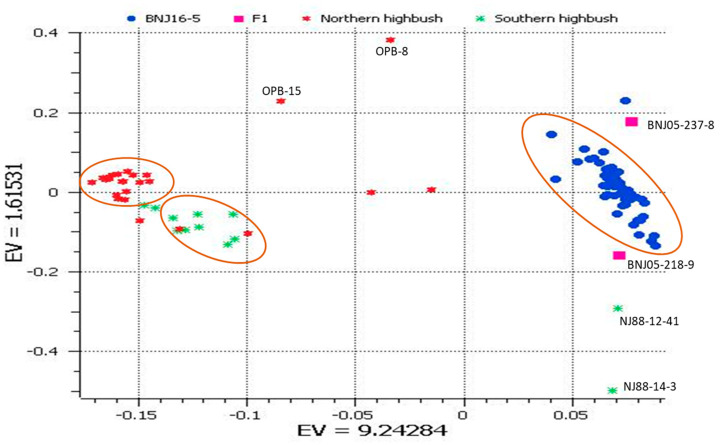
The first and second components of principal component analysis for 99 blueberry accessions. See Table S3 for eigenvalues for the respective positions of individual plants in this figure.

We used PCA to distinguish closely related individuals in groups and to understand the genetic relatedness of blueberry cultivars used in the present study. The PCA with first and second eigenvectors explained 10.8% of the total variance (Figure 1). From the results, three main groups were identified, including (1) NHB cultivars, (2) SHB cultivars, and (3) F_1_ and F_2_ progenies of the cross BNJ16-5. The F_1_ and F_2_ plants and parents were distinguished, and all progenies were placed in between the parental lines. This observation suggested that the population can be explored for admixture analysis. The eigenvalues of the first two principal components for all blueberry accessions used in this analysis are given in Table S3. The NHB group comprised 25 blueberry cultivars, of which two accessions (NJOPB-8, and NJOPB-15) were diploid *V. corymbosum*. Most of the NHB types grouped closely on PCA, but five cultivars were diverse, along with the two diploid and wild *V. corymbosum* accessions at a distance from the main cluster (Figure 1). The NHB cultivars ‘G-751’, ‘Sweetheart’, and ‘Pink Lemonade’ grouped close to the SHB group, thereby suggesting admixture. We do not know the pedigree backgrounds of ‘G-751’ and ‘Sweetheart’. ‘Pink Lemonade’ is a hybrid derivative of *V. corymbosum* and rabbiteye blueberry (cross of NJ89-158-1 x Delite (*V. ashei*)).

### 2.2. Admixture Analysis

**Figure 2:**
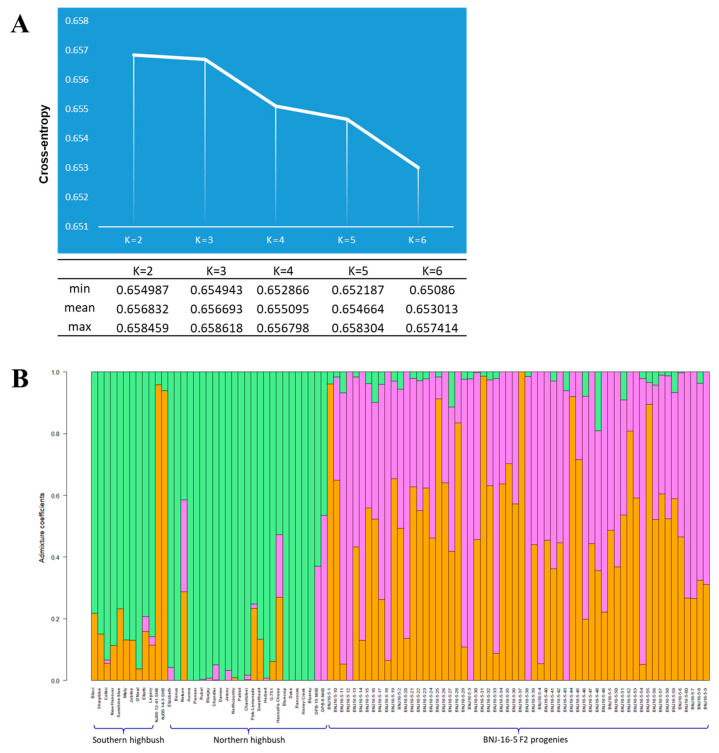
(**A**) Detection of the number of clusters (K) based on the cross-entropy criterion by LEA software. The selected value in this analysis was K = 3, for which the cross-entropy curve exhibits a plateau. (**B**) Admixture analysis of the blueberry accessions comprising northern and southern highbush types and F_2_ progenies. Each individual is represented by a vertical line depicting its membership into two clusters: orange bars are with a diploid *V. darrowii* background and magenta bars are with a diploid *V. corymbosum* background.

To resolve lineage sorting of tetraploid cultivated genomes, we used admixture analysis with the Landscape and Ecological Association model [33], which chooses a cross-entropy criterion (prediction of a fraction of masked genotypes (matrix completion)). We iterated six runs (K) and chose K = 3 because the value of K-3 showed a plateau of cross-entropy curve indicating a statistically significant lineage pattern (Figure 2A). From this analysis, the cultivars were admixed with three lineages (Figure 2B). Of note, 10 of 25 NHB cultivars had no admixture. ‘Honey Creek’, ‘Blueray’, ‘Bonus’, ‘Aurora’, ‘Pioneer’, ‘Darrow’, ‘Patriot’, ‘Bluecrop’, ‘Duke’, and ‘Rancocas’ had a single lineage, and also were closely grouped (shown in orange color) on PCA. In contrast, SHB cultivars were highly admixed. The genetically diverse SHB group in this study consisted of 9 tetraploids and a diploid genotype *V. elliottii*. The NJ88-12-41 and NJ88-14-3 were *V. darrowii* genotypes used as parents for the development of BNJ16-5 progenies. The admixture coefficients for SHB cultivars were in the range of 0.02 to 0.26, but those for the diploid *V. darrowii* species NJ88-14-3 and NJ88-12-41 were 0.93 and 0.95.

Admixture analysis to reveal the parental lineage is also significant in progenies of an interspecific cross in a breeding program and can be helpful in the individual selection process. Hence, we explored progenies of an interspecific cross of *V. corymbosum* (NJOPB-8, and NJOPB-15) and *V. darrowii* (NJ88-14-3, and NJ88-12-41) to help understand the parental lineage distribution in the F_2_ generation. The distribution of genomic proportions based on admixture coefficients of *V. darrowii* (represented by orange color) and *V. corymbosum* (represented by magenta color) across the progenies are shown in Figure 2B. Genetic lineage distribution by admixture analysis of the 60 F_2_ progeny derived from a cross of two species *V. corymbosum* and *V*. *darrowii* was overall 50%*,* with wide variation for the admixture coefficients (from 0 to 1). For eight of these progenies, the admixture coefficients were <0.2 lineage from *V. darrowii* and could be promising to select for lines with little introgression from wild materials. Of the progenies, BNJ16-5-4, BNJ16-5-11, BNJ16-5-18, and BNJ16-5-33 had <10% lineage from *V. darrowii*. In contrast, BNJ16-5-25, BNJ16-5-44, and BNJ16-5-55 had <10% lineage from *V. corymbosum*. Most of the cultivated NHB species are preferred for commercial production because of desirable fruit and horticultural traits but, owing to high chilling requirements, are confined to colder environments. However, southern species require fewer chilling hours than do northern species, and SHB cultivars may have acquired heat tolerance from *V. darrowii*. Admixture analysis in this study identified progenies with <10% parental lineage from either species, which can be of great importance in breeding blueberries with desirable traits, including chilling hour requirements, and fruit composition.

### 2.3. F_ST_ for Characterizing Selection Footprints

**Figure 3:**
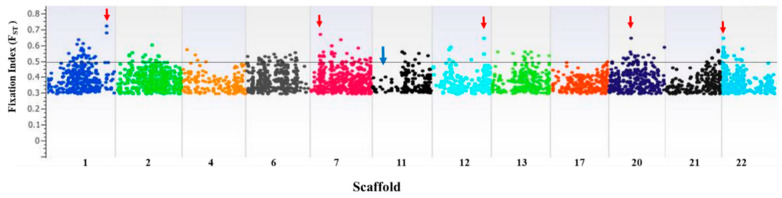
Genome-wide window-based pairwise fixation index *F_ST_* values for northern and southern highbush blueberry accessions across the 12 scaffolds. Red arrows indicate markers with high *F_ST_* values. Blue arrow on part of scaffold 11 shows a distinct sweep. See Table S4 for *F_ST_* values.

We used *F_ST_* analysis, estimating with 95% confidence intervals (CIs), to identify chromosomal regions that genetically distinguish NHB and SHB cultivars used in the analysis. The overall *F_ST_* value between the NHB and SHB cultivars was 0.0473, which indicates wide genetic differentiation between these two highbush-type cultivars. The *F_ST_* distribution is shown in a Manhattan plot for all scaffolds showing regions with high *F_ST_* markers (Figure 3). Pairwise *F_ST_* values across all scaffolds are in Table S4. A wide sweep on scaffold 11 was noted and was also characterized by decreased nucleotide diversity. From high pairwise *F_ST_* indices, regions that underwent positive selection are noted with the arrow marks in the Manhattan plot; they contributed to the divergence of NHB from SHB cultivar groups (Figure 3) and could be important for genetic improvement.

### 2.4. Nucleotide Diversity

**Figure 4:**
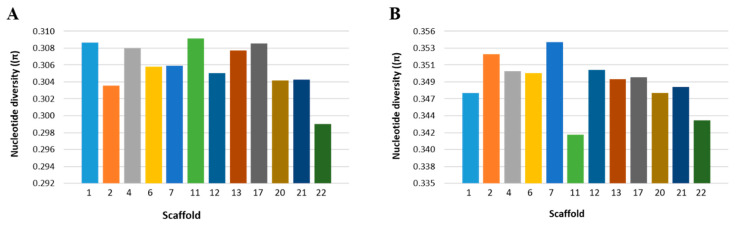
Scaffold-wise frequency spectrum of nucleotide diversity (π) in northern (**A**) and southern (**B**) highbush blueberry accessions.

We estimated nucleotide diversity (π) across the 12 scaffolds to assess the patterns of diversity among the NHB and SHB cultivars used in this study (Figure 4). Scaffold-wise, nucleotide diversity of NHB cultivars ranged from 0.29 to 0.31 but from 0.34 to 0.35 for SHB cultivars (Table S5).

### 2.5. LD and Haplotype Analysis

**Figure 5:**
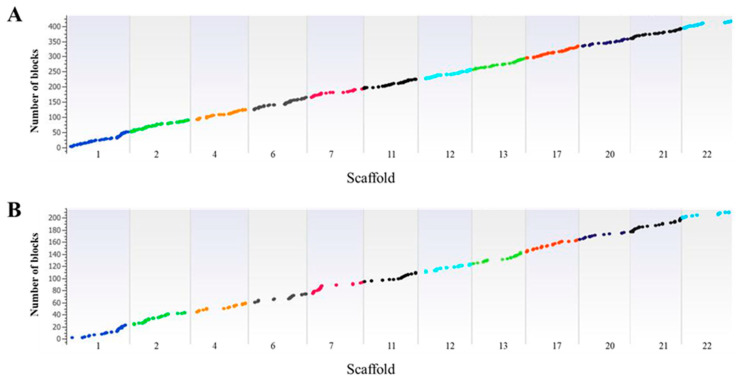
Haplotype distribution across the 12 scaffolds for (**A**) northern and (**B**) southern highbush blueberry accessions. A total of 416 haplotype blocks with 993 SNPs were identified in northern highbush genotypes, with 209 haplotype blocks and 578 SNPs identified in southern highbush genotypes. See Tables S6 and S7 for detailed haplotype information.

**Figure 6:**
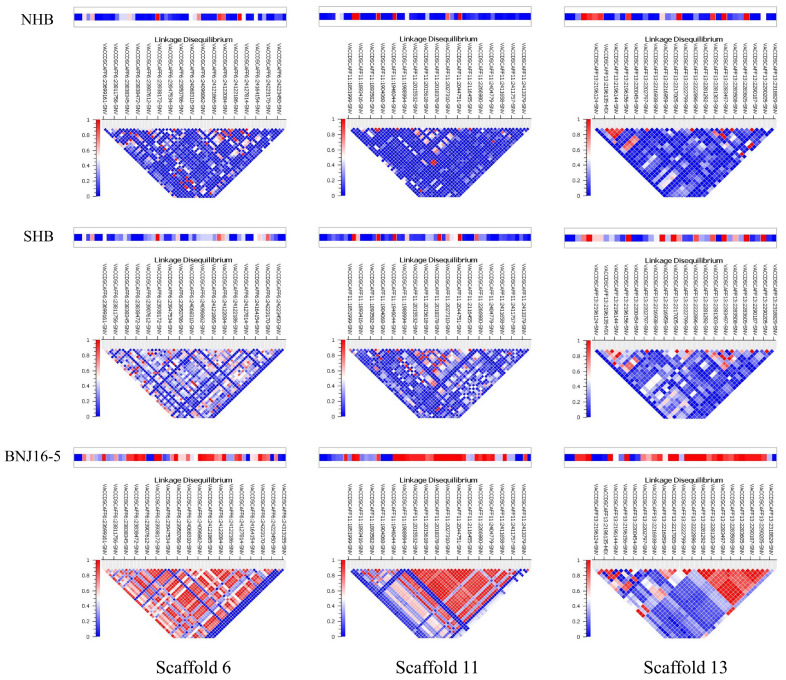
Comparative analysis of the linkage disequilibrium (LD) blocks across three scaffold regions in northern highbush (NHB), southern highbush (SHB) and BNJ 16-5 populations. Red-colored block indicates the highest LD and blue-colored blocks indicate the lowest LD values.

LD is the non-random association of alleles between different loci and is affected by several factors including recombination rate, population structure, and genetic linkage. To understand the extent of genetic variation patterns, we performed an extensive LD block analysis of NHB and SHB cultivars. The largest LD blocks across the scaffolds were estimated (Table 2). In NHB genotypes, the largest LD block was 672 kb on scaffold 13, whereas the largest LD block in SHB genotypes was 425 kb. The size and number of haplotypes and their distribution are presented in Manhattan plots (Figure 5). For NHB genotypes, we identified 416 haplotype blocks with 993 SNPs ranging from 4 to 10 SNPs per haplotype, whereas for SHB genotypes, we identified 209 haplotype blocks with 578 SNPs ranging from 2 to 12 SNPs per haplotype (Tables S6 and S7). The average number of SNPs in the haplotype blocks was higher in SHB than NHB cultivars (2.73 vs. 2.39). LD was estimated by using adjacent SNPs within a scaffold to reduce spurious associations. Highly significant LD blocks in the NHB and SHB genotypes and the BNJ16-5 population are shown in Figure 6 for comparison. Cross derivatives of the BNJ16-5 population showed a range of LD decays among the scaffolds, indicating variable recombination rates across scaffolds.

## 3. Discussion

Blueberry cultivars are derivatives of complex interspecific crosses involving four different species [25]. This situation warrants performing admixture analyses of progenies to understand lineage sorting primarily to classify them into NHB and SHB breeding material. This analysis will significantly reduce the time required in identifying progenies based on phenotype selections, the main selection criteria in traditional development programs. The PCA and admixture analysis in this study revealed a wide distribution of parental lineages with complex genetic makeup, which can be helpful for improvement.

To our knowledge, this is the first study in which the SNPs identified by using GBS were aligned to the tetraploid *V. corymbosum* cv. Draper v1.0 reference genome sequence [32] and made available for public use. In this study, we mapped 2.8 million reads to the reference genome, which corresponded to an overall 83% mapping to the genome. A stringent filtering with MAF = 0.05 and 90% call rate yielded 92,048 SNPs. Furthermore, genome-wide LD blocks and haplotypes were characterized for comparing NHB and SHB cultivars.

NHB blueberries are the most frequently cultivated species because of their high fruit quality and resistance to low temperatures [34]. NHB cultivars are reported to have significantly greater levels of anthocyanidins as compared with the other varieties. SHB cultivars were developed by further introgression of *V. darrowii* and other southern species in *V. corymbosum* background. They combine nutritional benefits from the northern blueberries and low chilling requirements of the southern blueberries, which are adaptive to southern growing regions. Recombination between *V. corymbosum* and *V*. *darrowii* genomes is apparent in the F_1_ parents although LD appears more prevalent in some regions than others. Desirable traits linked to undesirable traits in these regions may suffer linkage drag in the breeding process. There is also the possibility of recombination being reduced between interspecific genomes in polyploids if preferential pairing occurs. Our PCA revealed three NHB cultivars positioned close to the SHB group. Previous studies by Boches et al. [19] and Zong et al. [27] observed similar overlapping of the NHB and SHB cultivars, owing to the resemblance in genetic backgrounds. Such shared germplasm sources of NHB and SHB can be of immense use for introgression of nutraceutically important traits as well as stress tolerance.

Blueberry domestication is relatively a recent event, initiated by Elizabeth White, a horticulturist for a private company in New Jersey (NJ, United States), and Frederick Coville, who was the chief botanist of the United States Department of Agriculture [35]. The germplasm selections from this project laid the foundation for modern plant breeding programs to develop improved varieties for commercial cultivation [36]. The useful characteristics in diploid-section *Cyanococcus* species and the existence of key commercial cultivars at both the tetraploid and hexaploid levels helped blueberry breeders perform successful gene introgressions in tetraploid and hexaploid blueberries [37]. Such multiple interspecific crosses within *Vaccinium* species enabled genetic gain for increased fruit size and yield and also expanded the geographic limits of highbush blueberry production. Highbush blueberries were domesticated because the domesticated plants produced larger, more uniform fruit, and in a higher quantity than their diploid wild ancestors [38,39,40]. These programs significantly widened the genetic diversity between the domesticated cultivars and diploid wild progenitors with a *V. corymbosum* background [23] and increased the genetic distance between diploid and polyploidy.

In this study, we offered insight into the genome-wide differences between cultivars and wild blueberry diploid *V. corymbosum* and *V. darrowii* accessions and their cross derivatives. On PCA plot, SHB and NHB cultivar groups were positioned adjacent to each other because the NHB *V. corymbosum* genome was a common background. However, diploid wild accessions of *V. corymbosum* and *V. darrowii* were positioned further away from SHB and NHB cultivar groups. Our results indicated a wide genetic divergence between the blueberry cultivar groups and wild diploid accessions. Because the origin of 4x NHB from the putative progenitor diploid *V. corymbosum* is further supported by fruit chemistry [31], the observed variation may be sympatric, involving complex genetic processes underlying ploidy. Along similar lines, Wang et al. [31] found tetraploid (NHB) versus diploid *V. corymbosum* to be divergent for flavonol aglycone and glycosylation composition. It would appear that germplasm at the diploid level has much to offer breeding programs. Similar results were also described by Mengist et al. [41]. A significant proportion of genetic variation among the clones was documented in earlier studies [42].

Our analysis showed that 10 of 25 NHB cultivars featured no admixture. Many of these cultivars are known to have 3.1% to 28% *V. angustifolium* background [25]. ‘Bluecrop’ and ‘Blueray’ were released around the 1950s and were known to have derived from the same parentage. The common background of these two cultivars was previously confirmed by Zong et al. [27]. The remaining NHB cultivars (‘Nelson’, ‘Hannah’s Choice’, ‘Pink Lemonade’, ‘Sweetheart’, ‘G-751’, and the two diploid wild accessions NJOPB-8 and NJOPB-15) had lesser admixture coefficients in this study. Two NHB cultivars, ‘Nelson’ and ‘Hannah’s Choice’, did not cluster with the NHB cluster. ‘Pink Lemonade’, ‘Sweetheart’, and ‘G-751’ were clustered close to the SHB group on PCA. Current admixture analysis confirms that ‘Bluecrop’ is the parent of ‘Nelson’, as reported earlier [19,25]. Except for a few minor variations, clustering in the PCA was largely corroborated by the results of the admixture analysis.

The introgression of genes from undomesticated materials such as *V. darrowii* and *V. tenellum* into the SHB cultivars caused higher genetic diversity and expanded the geographic limits of the production of highbush blueberry (*V. corymbosum*) [43]. However, such introgressions also carried linkage drag, which could be very high in some SHB cultivars and affect agriculturally important traits. The hybrids between tetraploid *V. corymbosum* (CCCC) and diploid *V. darrowii* (DD) were most likely derived from 2*n* gametes from *V. darrowii* [37], giving a CCDD genomic composition in the hybrid. Preferential pairing in the polyploid of more homologous genomes would lead to linkage drag. Unfortunately, the unavailability of high-throughput screening methods may have restricted progeny selections. Admixture analyses using bi-allelic markers such as SNPs, which are spread all over the genome and are ubiquitous, can help to reveal the proportion of lineages from the parental lines used in various development programs.

Fruit quality, tolerance to high soil pH and mineral soils, chilling requirement, and cold-hardiness have been identified as important traits in blueberry. Of these, low chilling hour requirements, and better fruit composition can be the two crucial traits in ideotype selection. Introgressions from wild blueberry species have been used to transfer desired traits into a *V. corymbosum* background. Thus, interspecific hybridizations between *V. corymbosum* and *V. darrowii* have produced blueberry cultivars with improved fruit quality, low chilling hours, and resistance to stress (biotic as well as abiotic) [44]. However, we must understand the parental lineage distribution in the interspecific hybridizations to minimize the unwanted linkage drag. From the F_2_ progenies used in this study, BNJ16-5-4, BNJ16-5-11, BNJ16-5-18, and BNJ16-5-33 have <10% parental lineage from *V. darrowii*. Similarly, BNJ16-5-25, BNJ16-5-44, and BNJ16-5-55 have <10% parental lineage from *V. corymbosum*. These plants can be tested for their adaptability in climates with varying chilling hour conditions and may be promising lines for cultivar development. In this way, admixture analysis helps identify the exact proportions of the introgressions from each parental lineage, thereby helping in the selection process. It can further help improve the specific trait performance, as desired in blueberry ideotype breeding.

## 4. Materials and Methods

### 4.1. Plant Materials

In this study, we used 99 blueberry accessions, including 23 tetraploid NHB, 9 tetraploid SHB cultivars, and 1 diploid SHB cultivar (Table 3). Of the highbush cultivars, 26 were from the blueberry inventory maintained at Delaware State University, and 7 were provided by Dr. Nicholi Vorsa (the Philip E. Marucci Center for Blueberry and Cranberry Research, Chatsworth, NJ, USA). Undomesticated diploid germplasm included *V. darrowii* grandparents (NJ88-14-3, NJ88-12-41), *V. corymbosum* grandparents (NJOPB-8, NJOPB-15), F_1_ progeny (BNJ05-237-8 [NJOPB-8 × NJ88-12-41], BNJ05-218-9 [NJ88-14-3 × NJOPB-15]), and 60 F_2_ BNJ1-5 progeny (BNJ05-237-8 × BNJ05-218-9) of the cross *V. corymbosum* × *V*. *darrowii.* (Table 3). Note: BNJ05-237-8 was in *V. corymbosum* cytoplasm and BNJ05-218-9 in *V. darrowii* cytoplasm.

### 4.2. DNA Isolation

Leaf samples from young actively growing blueberry plants were collected in dry ice and stored at −80 °C. About 100 mg leaf tissue was placed in a 2-mL round-bottom tube containing a single 5-mm stainless steel bead. The tubes containing the leaf samples were frozen in liquid nitrogen for 5 min and homogenized by using TissueLyser II (Qiagen, Germantown, MD, USA) for two 3-min bursts at a frequency of 25 Hz. The homogenized leaf samples were stored at −80 °C. The DNA extraction was performed with a commercially available Plant DNA extraction kit (DNeasy mini plant kit, Qiagen, Germantown, MD, USA) following the manufacturer’s protocol with slight modifications. DNA was initially quantified by measuring absorbance at 260 nm by using NanoDrop 2000 (Thermo Fisher Scientific, Waltham, MA, USA). To prepare the samples for GBS, DNA samples with better quality were quantified by using the Qubit dsDNA HS Assay Kit (Thermo Fisher Scientific, Waltham, MA, USA) and measured by using the Qubit Fluorometer (Thermo Fisher Scientific, Waltham, MA, USA) and used for GBS library preparation. Sequencing was carried out at the Department of Biology, West Virginia State University, Institute, WA, USA.

### 4.3. GBS Analysis

We genotyped 99 blueberry samples by using the GBS technology for variant identification and GWAS analysis. GBS was performed as described [45]. Genomic DNA was digested by using the *Ape*K1 restriction enzyme and ligated with barcoded adapters. The adapter-ligated library from each sample was pooled and amplified with Illumina sequencing primers. The quality and quantity of the GBS library was assessed by using Bioanalyzer 2100 (Thermo Fisher Scientific, Waltham, MA, USA) and Qubit 4 fluorimeter (Thermo Fisher Scientific, Waltham, MA, USA). The library was sequenced by using the NextSeq500 platform with paired-end sequencing chemistry. The resulting image files in bcl format were converted to FASTQ with 2 × 75 bp reads by using bcl2fastq (Illumina, San Diego, CA, USA). The GBS reads were de-multiplexed and variants were called by using a new workflow with GB-eaSy (https://github.com/dpwickland/GB-easy), which has an advantage of using paired-end reads from GBS data to call variants [46]. The resulting variant call file (vcf) was used for further downstream analysis.

### 4.4. Sequence Alignment and SNP Identification

Sequencing reads were aligned to the *V. corymbosum* cv. Draper (tetraploid) v1.0 genome sequence [32]. The assembled genome data were downloaded from GigaDB (http://gigadb.org/dataset/100537). The longest 12 scaffold sequences were used to align the sequencing reads. The mapped GBS reads were used to call SNPs by using GB-eaSy.

### 4.5. Principal Component Analysis

The SNPs with MAF ≥1% and missing data (call) rate ≤90% were used for analyses. For analyzing population structure, we used principal components, or eigenvectors, of PCA, and corresponding eigenvalues were estimated by using the EIGENSTRAT algorithm [47] with the SNP and Variation Suite (SVS v8.8.5; Golden Helix, Inc., Bozeman, MT, USA, www.goldenhelix.com).

### 4.6. Admixture Analysis

Admixture was analyzed by using a least-squares optimization approach implemented in the sNMF function of the R package LEA [33,48]. This approach is based on estimating admixture coefficients based on sparse non-negative matrix factorization. The number of K populations was assessed from 1 to 6 clusters, and 10 replications were performed for each K value. The best K value was selected based on the minimum value of the cross-entropy criterion [48].

### 4.7. Haplotype Block Analysis

For GBS data, we considered only SNPs successfully mapped to the whole-genome sequence draft, because knowing the physical location of SNPs helps prevent spurious LD and, thereby, calling unreliable haplotype blocks. Mapped SNPs were further filtered by call rate >90%. Before studying LD decay, haplotype blocks were calculated for all markers by using the default settings in SVS v8.8.5. (Golden Helix, Inc., Bozeman, MT, USA, www.goldenhelix.com). Adjacent and pairwise measurements of LD for GBS data were calculated separately for SNPs in each scaffold. All LD plots and LD measurements and haplotype frequency calculations involved using SVS v8.8.5 (Golden Helix, Inc., Bozeman, MT, USA, www.goldenhelix.com).

### 4.8. Nucleotide Diversity Analysis

Expected nucleotide diversity (π) for various chromosomes were estimated with sliding-window analysis by using TASSEL v5.0 as described [49]. Estimation of fixation index (*F_ST_*) was based on Wright’s F statistic [50] with use of SVS v8.8.5 (Golden Helix, Inc., Bozeman, MT, USA, www.goldenhelix.com).

## 5. Conclusions

In this study, we have shown the efficiency of GBS with a single restriction enzyme *Ape*K1 in generating high-density genotype data for genetic diversity and admixture analyses in blueberry. We successfully mapped the GBS-obtained sequence reads to the genome sequence of the tetraploid variety Draper, and the identified SNPs were used in PCA, haplotype, and admixture analysis to understand genetic relatedness in blueberry accessions. With goals to improve the adaptability of blueberries to wider geographies and warmer climates, interspecific hybridizations within *Vaccinium* species are set to increase greatly. In such a scenario, it will be highly crucial to resolve the genomic contribution of the two parental species in hybrid progenies. Admixture analysis of progenies by using high-throughput SNP markers distributed across chromosomes will be useful to reveal genetic lineages. Our study showed how genetic admixture analysis is accurate for selecting progenies with desired parental lineage in intercross populations.

## Figures and Tables

**Table 1:** Scaffold-wise summary of the single-nucleotide polymorphism (SNP) statistics from the genotyping-by-sequencing analysis across 99 blueberry accessions.

Chromosome	Chromosome Length (bp)	Raw SNPs	Filtered SNPs (MAF < 0.05; Call Rate < 0.9; DP > 3)	Average Number of Filtered SNPs per kb
VACCDSCAFF1	46,295,995	223,567	8719	5
VACCDSCAFF2	44,818,276	188,522	8994	5
VACCDSCAFF4	42,981,373	162,229	7758	6
VACCDSCAFF6	42,795,824	190,595	7090	6
VACCDSCAFF7	41,705,179	175,039	7783	5
VACCDSCAFF11	40,122,599	211,211	8194	5
VACCDSCAFF12	39,741,682	170,459	6191	6
VACCDSCAFF13	39,652,356	177,072	7654	5
VACCDSCAFF17	38,874,919	173,797	7901	5
VACCDSCAFF20	37,996,905	187,631	7116	5
VACCDSCAFF21	37,975,728	184,224	7395	5
VACCDSCAFF22	37,315,645	199,693	7253	5
Total Number of SNPs		2,244,039	92,048	

**Table 2:** Scaffold-wise distribution of linkage disequilibrium (LD) blocks for northern and southern highbush cultivars used in this analysis.

Chromosome	Largest LD Block (kb)	
	Northern Highbush Blueberry	Southern Highbush Blueberry
VACCDSCAFF1	285	285
VACCDSCAFF2	425	425
VACCDSCAFF4	425	425
VACCDSCAFF6	518	418
VACCDSCAFF7	154	251
VACCDSCAFF11	255	329
VACCDSCAFF12	454	382
VACCDSCAFF13	672	261
VACCDSCAFF17	545	243
VACCDSCAFF20	247	255
VACCDSCAFF21	231	366
VACCDSCAFF22	189	194

**Table 3:** List of northern and southern blueberry cultivars and cross derivatives used in the present work.

Category	Genotype	Accession ^a^	Ploidy	Taxon	Pedigree	Improvement Status
Tetraploid northern highbush (23) ^b^	Elizabeth		4×	*Vc*	(Katharine × Jersey) × Scammel	
	Bonus	PI 666839	4×	*Vc*		
	Nelson	PI 618100	4×	*Vc*	Bluecrop × G-107	Cultivar
	Aurora		4×	*Vc*	Brigitta Blue × Elliott	
	Pioneer	PI 554815	4×	*Vc*	Brooks × Sooy	Cultivar
	Rubel	PI 554817	4×	*Vc*	Selection from wild *V. corymbosum* in NJ selected from the pine barrens of NJ	Cultivar
	Bluejay	PI 554846	4×	*Vc*	Berkeley × Michigan Highbush Sel. 241 (Pioneer × Taylor)	Cultivar
	Chandler	PI 657260	4×	*Vc*	Darrow × M-23	Cultivar
	Darrow	PI 618035	4×	*Vc*	F 72 × Bluecrop	Cultivar
	Jersey	PI 554897	4×	*Vc*	Rubel × Grover	Cultivar
	Northcountry	PI 554953	4×	*Vc × Va*	B6 (G65 × ‘Ashworth’ *V. corymbosum*) × R2P4 (open pollinated *V. corymbosum* × *V. angustifolium* hybrid)	Cultivar
	Patriot	PI 554843	4×	*Vc*	US 3 (Dixi × Mich LB-1) × Earliblue	Cultivar
	Chanticleer	PI 638765	4×	*Vc*	G-180 × MEUS 6620	Cultivar
	Pink Lemonade	PI 641330	4×	*Vc*	NJ89-158-1 × Delite (*V. ashei*)	Cultivar
	Sweetheart		4×	*Vc*		Cultivar
	Herbert	PI 554805	4×	*Vc*	Stanley (Katharine × Rubel) × GS-149 (Jersey × Pioneer)	Cultivar
	G-751		4×	*Vc*		Wild material
	Hannah’s Choice	PI 657259	4×	*Vc*	G-136 × G-358	Cultivar
	Bluecrop	PI 554885	4×	*Vc*	GM-37 (Jersey × Pioneer) × CU-5 (Stanley × June)	Cultivar
	Duke	PI 554872	4×	*Vc*	G-100 (Ivanhoe × Earliblue) × 192-8 (E-30 × E-11)	Cultivar
	Rancocas	PI 554816	4×	*Vc*	394Y (Brooks × Russell) × Rubel	Cultivar
	Honey Creek		4×	*Vc*		
	Blueray	PI 554887	4×	*Vc*	(Jersey × Pioneer) × (Stanley × June)	
Tetraploid southern highbush (9)	Biloxi	PI 618193	4×	*Vc*	Sharpblue × US 329 [US210 (US67 × US132) × FL 4-76 (Bluecrop × 13-236)]	Cultivar
	Sharpblue	PI 554948	4×	*Vc*	*V. corymbosum* × *V. ashei* & *V. darrowii* (Fla 61-5 × Fla 62-4) tetraploid	Cultivar
	Colibri		4×	*Vc*		
	New Hanover		4×	*Vc*		
	Sunshine blue	PI 555316		*Vc × Va*	Avonblue OP	
	Misty	PI 555317	4×	*Vc*	Florida 67-I × Avonblue	
	Jubilee	PI 618195	4×	*Vc*	Sharpblue × MS60 [(Ashworth × Earliblue] × Bluecrop) × US-75]	Cultivar
	O’Neal	PI 554944	4×	*Vc*	Wolcott x Fla. 4-15 mainly *V. corymbosum*, some *V. angustifolium*, *V. ashei*, *V. darrowii*	Cultivar
	Legacy	PI 618164	4×	*Vc*	Elizabeth × (Fla. 4B × Bluecrop)	Cultivar
*Vaccinium elliottii* (1)	Elliottii	PI 657176	2×	*Ve*		Wild material
*Vaccinium darrowii* (2)	NJ88-12-41		2×	*Vd*		Wild material
	NJ88-14-3		2×	*Vd*		Wild material
*Vaccinium corymbosum* (2)	NJOPB-8		2×	*Vc*		Wild material
	NJOPB-15		2×	*Vc*		Wild material
F_1_ (2)	BNJ05-237-8			*Vc × Vd*		Cross derivative
	BNJ05-218-9			*Vd × Vc*		Cross derivative
F_2_ (60)	BNJ16-5 population					Cross derivatives

*Vc, Vaccinium corymbosum; Vd, Vaccinium darrowii; Va, Vaccinium angustifolium; Ve, Vaccinium elliottii*. ^a^ The accession numbers are taken from United States Department of Agriculture Germplasm Resource Information Network (USDA-GRIN) database (https://npgsweb.ars-grin.gov/gringlobal/search). ^b^ The number in the parenthesis indicates the total number of genotypes in the respective category/species.

## Data Availability

The raw paired-end Illumina sequencing reads generated in the current study are available in the Sequence Read Archive (SRA) at NCBI under the Bio project accession number PRJNA687760.

## Supplementary Materials

The following are available online at https://www.mdpi.com/1422-0067/22/1/163/s1. Table S1: GBS summary of all the blueberry genotypes and BNJ16-5 population. Table S2: Details of the SNPs mapped to the longest 12 scaffold sequences of Draper v1.0 genome. Table S3: Eigenvalues for the first three principal components estimated for NHB, SHB, and 16-5 populations. Table S4: Pairwise *F_ST_* values of NHB and SHB cultivars across all the scaffolds. Table S5: Nucleotide diversity indices for NHB and SHB cultivars used in this study; Table S6. Details of the haplotype block information of NHB cultivars across all the scaffolds; Table S7. Details of the haplotype block information of SHB cultivars across all the scaffolds.
